# *Pseudomonas oryzihabitans* D1-104/3 and *P. gessardii* C31-106/3 differentially modulate the antioxidative response of duckweed (*Lemna minor* L.) to salt stress

**DOI:** 10.3389/fmicb.2024.1481437

**Published:** 2024-12-18

**Authors:** Tatjana Popržen, Dragana Antonić Reljin, Branka Uzelac, Marija Milovančević, Danijela Paunović, Milana Trifunović-Momčilov, Marija Marković, Martin Raspor, Ivan Nikolić, Slaviša Stanković, Olga Radulović

**Affiliations:** ^1^Department of Plant Physiology, Institute for Biological Research “Siniša Stanković” – National Institute of the Republic of Serbia, University of Belgrade, Belgrade, Serbia; ^2^Center for Biological Control and Plant Growth Promotion, Faculty of Biology, University of Belgrade, Belgrade, Serbia

**Keywords:** antioxidant, bacteria, duckweed, plant-growth promotion, stress, tolerance

## Abstract

**Introduction:**

The common duckweed (*L. minor*) is a model organism for investigation of plant physiology, especially stress-related responses. Its two physiological characteristics are of special interest: (1) salt-stressed duckweeds may accumulate starch, a precursor for biofuel; (2) duckweeds are associated with various beneficial (plant-growth promoting, PGP) bacterial strains. In this paper, we analyzed the role of two bacterial strains: *Pseudomonas oryzihabitans* D1-104/3 and *P. gessardii* C31-106/3 in regulation of duckweed's growth and antioxidative responses to salt (10 and 100 mM NaCl) and hypothesized that they alleviate salt-induced oxidative stress.

**Methods:**

Fresh and dry weight, frond number, photosynthetic pigments, malondialdehyde (MDA) and hydrogen peroxide (H_2_O_2_), ascorbic acid (AsA), proline, total polyphenol (TPC) and starch content, as well as antioxidant capacity and antioxidant enzymatic activity were measured after 14 days. Fluorescence microscopy was used to visualize bacterial presence on duckweeds.

**Results:**

Fluorescence microscope revealed that *Pseudomonas* bacteria colonized all duckweed surfaces. The doubling time of duckweeds inoculated with *P. gessardii* C31-106/3 was significantly longer. Additionally, at 0 and 10 mM NaCl, this strain decreased chlorosis in duckweeds. Moreover, *P. gessardii* C31-106/3 increased dry-to-fresh-weight ratio, proline, chlorophyll a, b and carotenoid content at 100 mM, as well as AsA content in plants in NaCl-free medium, while *P. oryzihabitans* D1-104/3 increased AsA at 100 mM NaCl. Both bacterial strains decreased lipid peroxidation, while *P. gessardii* C31-106/3 increased and *P. oryzihabitans* D1-104/3 decreased H_2_O_2_ content at 100 mM and 0 mM NaCl, respectively. Bacteria significantly increased TPC and antioxidant capacity at 100 mM NaCl, particularly *P. oryzihabitans* D1-104/3. After 14 days, the SOD and POX activities were at the same level in all samples. At 100 mM NaCl, CAT activity was increased in all plants.

**Discussion:**

The results of this study show that two *Pseudomonas* strains had markedly different effects on duckweed: while *P. oryzihabitans* D1-104/3 supported growth, *P. gessardii* C31-106/3 prioritized salt stress tolerance in duckweeds.

## 1 Introduction

Abiotic stress has severe effects on plants worldwide, as it can spread faster and affect plants more negatively than biotic stresses (Meena et al., 2017). Due to climate change and anthropogenic factors (e.g., mining, mineral fertilizers, irrigation, water erosion, construction industry, and rising sea levels), plants are increasingly exposed to salt stress, leading to disturbances in biodiversity and significant annual yield losses (Isayenkov and Maathuis, 2019; El Moukhtari et al., 2020). Increased salinity has multiple negative effects on plants: osmotic imbalance; cytotoxicity caused by excess accumulation of Na^+^ and Cl^−^ ions; and finally, negative nutritional effects due to impaired biosynthesis (El Moukhtari et al., 2020; Ullah et al., 2021; Hui et al., 2023). Effects of salt stress progress through several stages over time: in early phases, the stomatal closure will prevent water loss. However, this leads to conditions that favorize accumulation of reactive oxygen species, ROS (Gamalero and Glick, 2022); To counteract this, plants will activate antioxidant enzymes and low-molecular weight non-enzymatic antioxidants such as ascorbic acid and proline (El Moukhtari et al., 2020; Chen et al., 2021). If the salt stress persists, the plants will mobilize long-term defense mechanisms, which depend on the crosstalk between abscisic acid, ethylene and indole-3-acetic acid (Razzaque et al., 2019; Gamalero and Glick, 2022). Only a small number (cca. 2%) of highly specialized plants i.e., the halophytes acquired a true tolerance to salinity during evolution: most plants are extremely sensitive to NaCl concentrations >100 mM (Ullah et al., 2021; Gamalero and Glick, 2022; Hui et al., 2023). Therefore, it is necessary to improve the resistance of plants to salt stress, preferably by using sustainable and cheap biotechnological solutions (Gamalero and Glick, 2022). The plant-growth promoting bacteria (PGPB) are a group of bacterial strains with the ability to alleviate effects of stress and increase the yield of important crops (Kumar et al., 2020; Gamalero and Glick, 2022). The PGPB mechanisms of action are multifold: regulating uptake of nutrients such as phosphorus, iron and nitrogen; fine-tuning phytohormone levels; removing 1-aminocyclopropane carboxylic acid (ACC), the precursor for ethylene; and secreting protective, osmoregulating substances, e.g., exopolysaccharide polimers (Ali et al., 2014; Meena et al., 2017). However, to survive abiotic stress, plants will slow down or stop their growth and re-direct this energy into long-term storage and synthesis of protective substances: the PGPB will enhance this effect (Isayenkov and Maathuis, 2019; Zboralski and Filion, 2023). Several studies with important crops reported that under basal conditions, the PGPB will stimulate vegetative growth (photosynthesis, nutrient uptake, leaf, root, and stem development) whose equivalent in duckweeds (Lemnaceae) is the multiplication or doubling (Ishizawa et al., 2019a,b; Kumar et al., 2020). The members of duckweed family apparently regulate salt stress responses differently, and even within the same species, different clones can have significantly dissimilar responses. However, the accumulation of starch seems to be a very common response to salt stress in duckweeds (Cheng, 2011; Sree et al., 2015; Ziegler et al., 2016; Appenroth et al., 2021; Ullah et al., 2021; Sree and Appenroth, 2022). Due to their ability to accumulate starch and double their biomass at a record time, the duckweeds are also considered as a useful source of raw material for the production of food and biofuel, e.g., bioethanol (Ishizawa et al., 2017a; Van Hoeck et al., 2015). The common duckweed (*Lemna minor*) is increasingly used in the studies of stress physiology in plants, due to its simplified morphology, vegetative reproduction, ability to grow in polluted or eutrophized water, and immunity to various phytopathogens. Salt stress experiments with duckweeds are also potentially useful for the study of physiology of plants under drought, since these two mechanisms overlap (Gamalero and Glick, 2022). In our previous study, we isolated and identified a bacterial collection from the root zone of the common duckweed (*L. minor*) which was predominantly inhabited by various *Pseudomonas* strains (Radulović et al., 2019). In our more recent studies of this bacterial collection, we described *Pseudomonas* strains with dual IAA-producing and IAA-degrading activity and plant-growth promoting traits, which prompted us to test their potential for application in different types of abiotic stress: in this case, the salt stress (Radulović et al., 2019; Popržen et al., 2023, 2024). Therefore, in this study we aimed to analyze the effects of salt stress at 10 and 100 mM NaCl on growth of our duckweed clone, as well as effects of two rhizosphere-associated strains *Pseudomonas oryzihabitans* D1-104/3 and *P. gessardii* C31-106/3 selected from our previous study due to their PGPB potential (Popržen et al., 2024) in alleviating salt stress effects on duckweeds.

## 2 Results

### 2.1 Visualization of interactions between duckweeds and *Pseudomonas* bacteria

**Figure 1:**
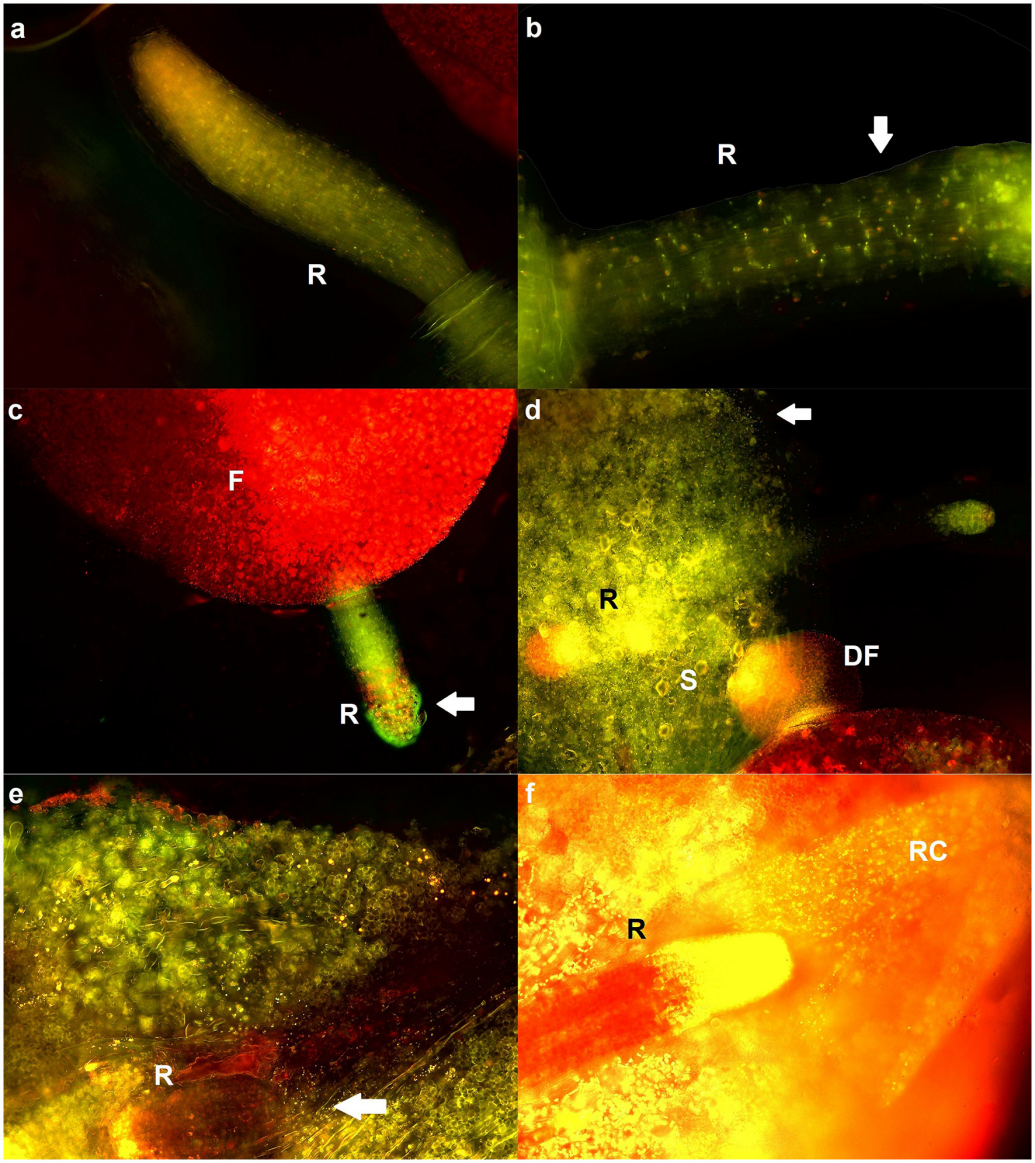
Representative fluorescent micrographs of duckweeds grown at 0, 10, and 100 mM NaCl. **(A)** Control (surface-sterilized duckweeds at 0 mM); **(B, D, F)** duckweeds inoculated with *P. oryzihabitans* D1-104/3 at 0, 10, and 100 mM NaCl; **(C, E)** duckweeds inoculated with *P. gessardii* C31-106/3 at 0 and 100 mM NaCl. Microcolonies are indicated with an arrow; F, frond; DF, daughter-frond; R, root; RC, root cap; S, stoma. Magnification: 20x **(A)**, 10x **(C)**, 40x **(B, D, E, F)**.

To analyze the localization of bacteria on surface of duckweeds, fluorescence microscopy was employed (Figure 1). After 14 days, the control emitted autofluorescence, but no bacteria were detected (Figure 1A). At the same time, microcolonies of both *Pseudomonas* strains were clearly visible on roots and fronds (Figures 1B–F). The signal coming from *Pseudomonas* bacteria was intense and more concentrated compared to autofluorescence of control plants, and their form was bacillar, in accordance with the expected shape of their microcolonies. On the roots, bacteria were the most visible in borders between root cells (Figure 1B) and on root caps (Figure 1F). High density of bacteria was observed on fronds, as well (Figures 1D, E). Most bacteria were alive, i.e., stained green (Figures 1B, D, E). Bacteria were found on live duckweeds and duckweeds in different stages of chlorosis, which emitted a red signal (Figures 1C, F). Stomata were clearly visible (Figure 1D).

### 2.2 Macroscopic parameters of duckweed growth exposed to salt stress and inoculated with *Pseudomonas* bacteria: doubling time, extent of chlorosis, and dry weight-to-fresh-weight ratio

**Figure 2:**
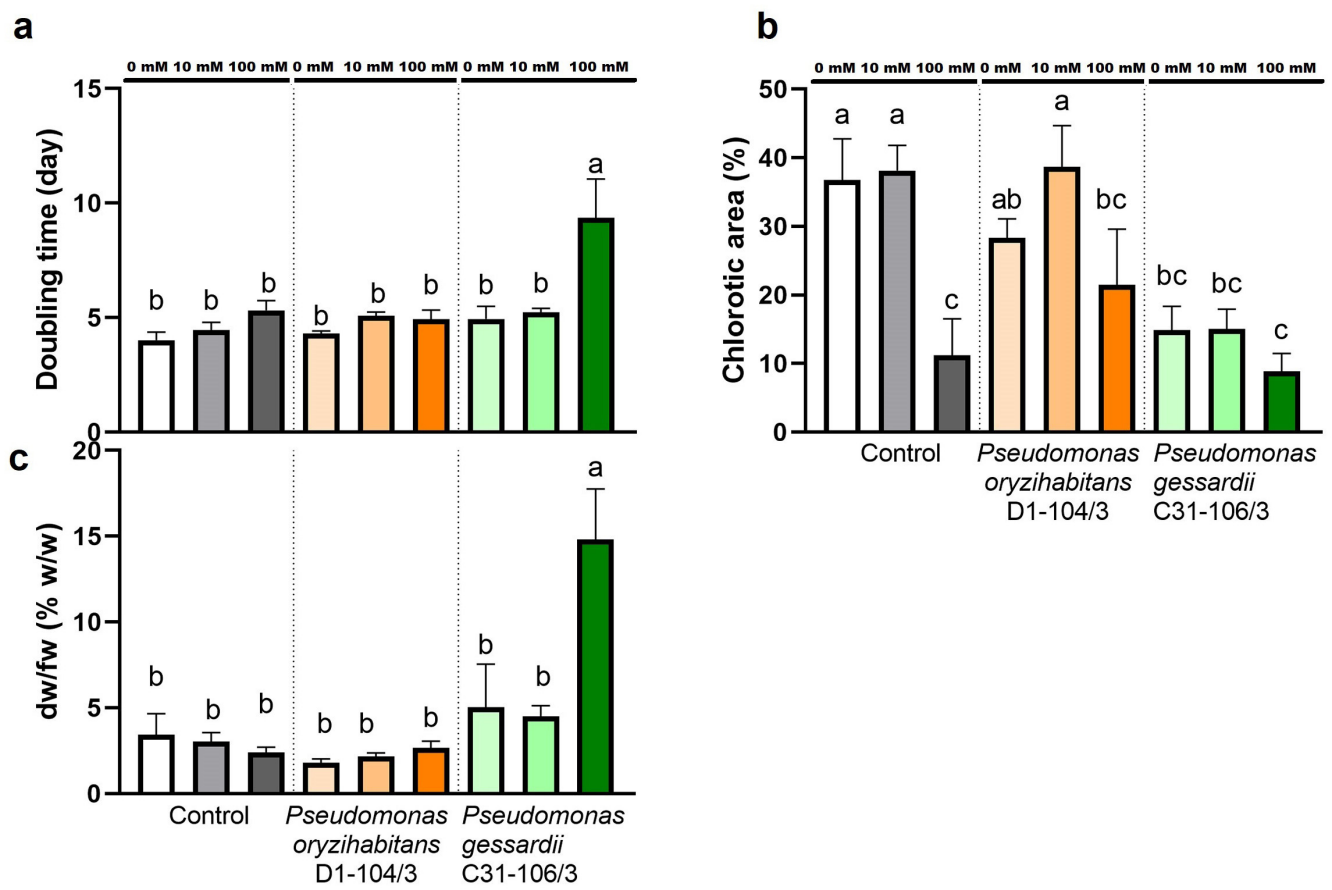
Macroscopic parameters of duckweeds' growth at 0, 10 and 100 mM NaCl. **(A)** doubling time (days); **(B)** chlorotic area (w/w %); **(C)** dry-weight-to-fresh-weight ratio (w/w %). Control—surface sterilized duckweeds. Orange columns—duckweeds inoculated with *P. oryzihabitans* D1-104/3. Green columns—duckweeds inoculated with *P. gessardii* C31-106/3. Bars represent the mean of three replicates, with standard errors. At least one same letter signifies no statistical difference (ANOVA, *p* < 0.05).

Macroscopic parameters of duckweed growth, namely: the doubling time, the extent of chlorosis, and the ratio of dry weight in fresh weight of duckweeds were evaluated in order to analyze the effects of salt treatment and *Pseudomonas* bacteria (Figure 2). To determine relative growth rates of duckweeds in this study, duckweeds were photographed after 14 days and the photographs were analyzed in *ImageJ*. Relative growth rates based on frond numbers were used to calculate doubling times (Figure 2A). Doubling times based on frond numbers were significantly longer at 100 mM, in duckweeds inoculated with *P. gessardii* C31-106/3.

After observing macroscopic differences in extent of chlorosis in duckweeds in this study, percentage of chlorotic area was estimated using *ImageJ*. At 0 and 10 mM NaCl, duckweeds inoculated with *P. gessardii* C31-106/3 were significantly less chlorotic than other two groups, i.e., there was ~50% less chlorosis in duckweeds inoculated with this bacterium (Figure 2B). At 100 mM NaCl, all plants showed approximately the same extent of chlorosis (ranging from 8 to 21.5%).

To estimate the dry weight content of duckweeds in this study, fresh duckweeds were oven-dried and their dry weight was measured. The results were presented as percentage of dry weight vs. fresh weight (% dw/fw) (Figure 2C). The results showed that duckweeds inoculated with *P. gessardii* C31-106/3 contained ~3 times more dry weight in the same amount of fresh weight than other specimens (Figure 2C).

### 2.3 Biochemical antioxidative and oxidative parameters of duckweeds exposed to salt stress and inoculated with *Pseudomonas* bacteria

#### 2.3.1 Non-enzymatic parameters: photosynthetic pigment content, lipid peroxidation, hydrogen peroxide, proline, ascorbic acid, total polyphenol content and antioxidant capacity, and starch content

##### 2.3.1.1 Photosynthetic pigment content

**Figure 3:**
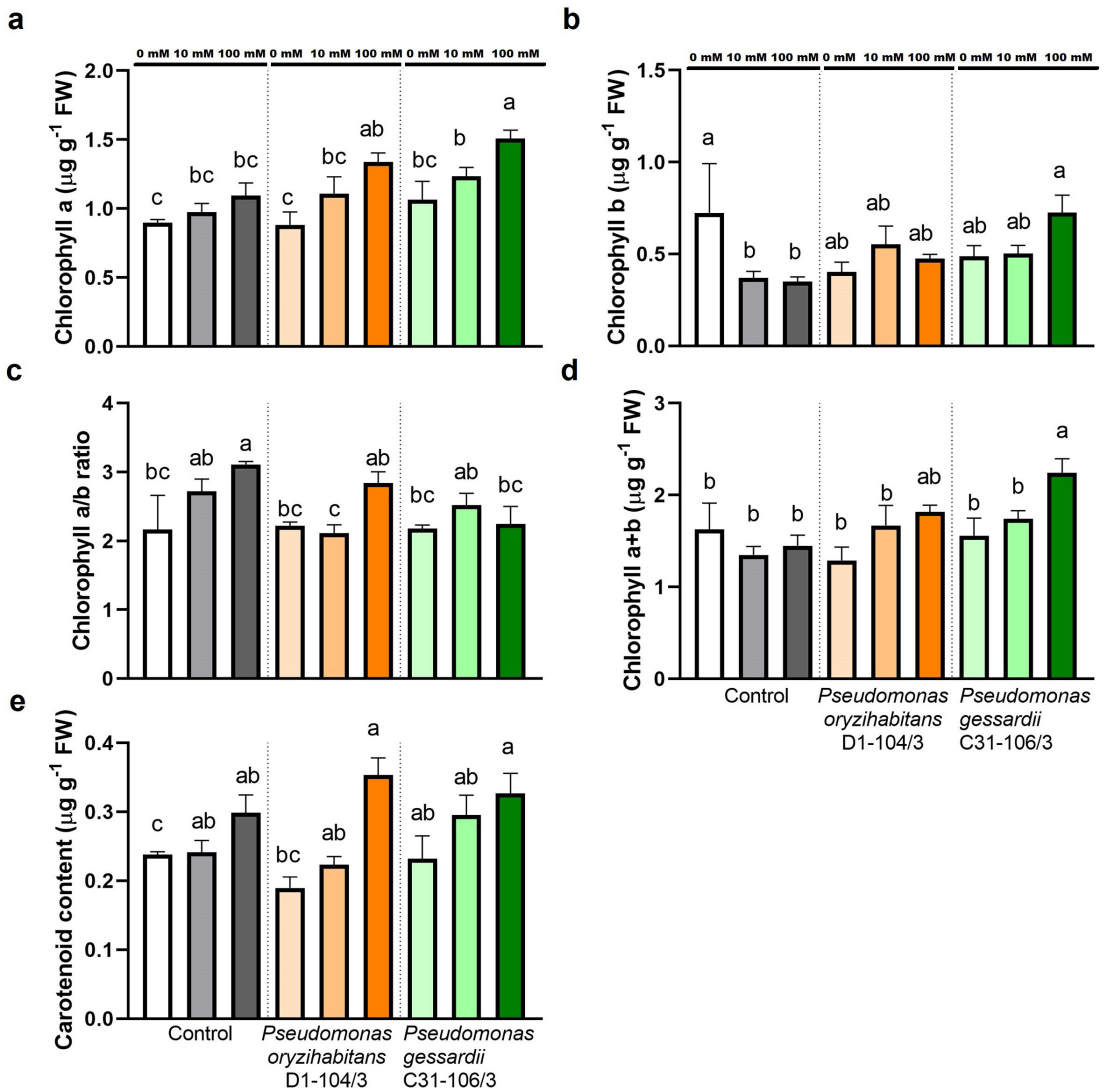
Photosynthetic pigment content of duckweeds at 0, 10 and 100 mM NaCl. **(A)** Chlorophyll a; **(B)** chlorophyll b; **(C)** chlorophyll a-to-b ratio; **(D)** chlorophyll a+b; **(E)** carotenoid. Control—surface sterilized duckweeds. Orange columns—duckweeds inoculated with *P. oryzihabitans* D1-104/3. Green columns—duckweeds inoculated with *P. gessardii* C31-106/3. Bars represent the mean of three replicates, with standard errors. At least one same letter signifies no statistical difference (ANOVA, *p* < 0.05).

Since photosynthetic pigments play a crucial role in antioxidative responses and are biomarkers of extent of oxidative stress, their content in fresh weight of duckweeds was determined (Figure 3). Chlorophyll a content was significantly increased in duckweeds inoculated with *P. gessardii* C31-106/3 at 100 mM NaCl (Figure 3A). At 100 mM NaCl, *P. gessardii* C31-106/3 significantly increased chlorophyll b content compared to surface-sterilized plants. The chlorophyll a/b ratio was the highest in surface-sterilized plants at 100 mM and the lowest in plants inoculated with *P. oryzihabitans* D1-104/3 at 10 mM (Figure 3C). Chlorophyll a + b (total chlorophyll) content was the highest in duckweeds inoculated with *P. gessardii* C31-106/3 at 100 mM (Figure 3D). Carotenoid content was mostly the same across treatments (Figure 3E).

##### 2.3.1.2 Lipid peroxidation and hydrogen peroxide content

**Figure 4:**
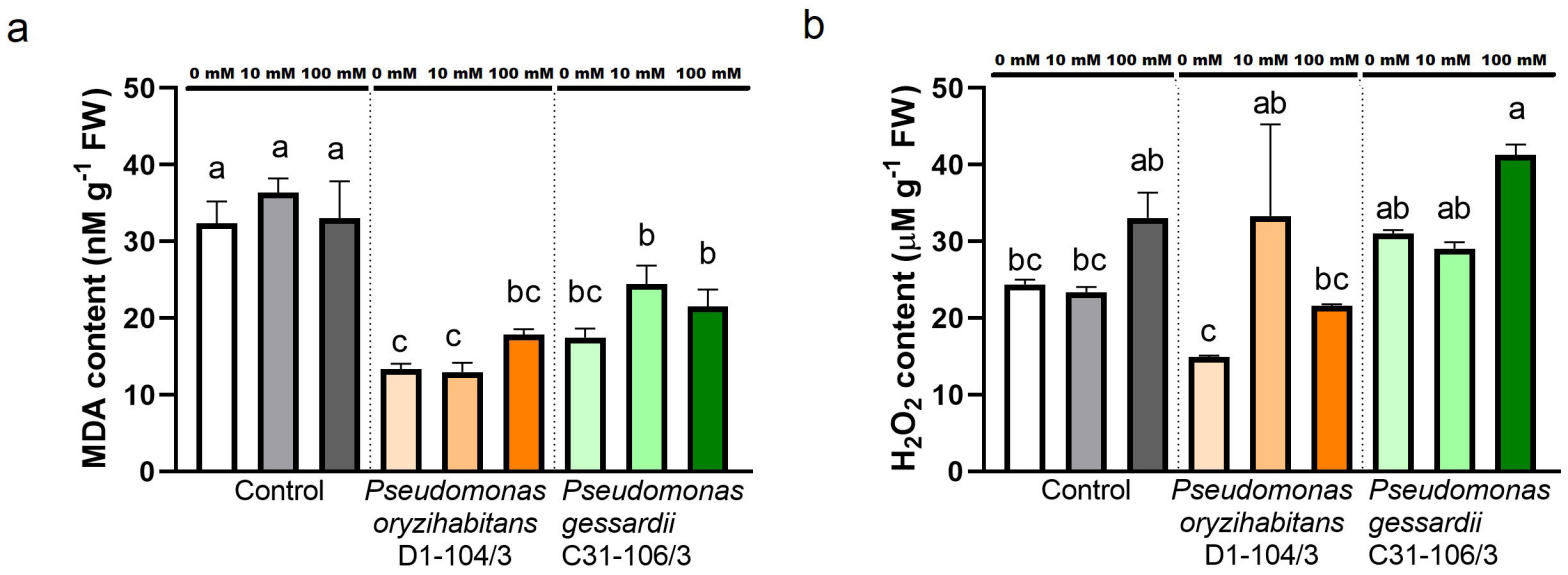
Lipid peroxidation and hydrogen peroxide content in duckweeds at 0, 10, and 100 mM NaCl. **(A)** Lipid peroxidation (MDA content); **(B)** H_2_O_2_ content. Orange columns—duckweeds inoculated with *P. oryzihabitans* D1-104/3. Green columns—duckweeds inoculated with *P. gessardii* C31-106/3. Bars represent the mean of three replicates, with standard errors. At least one same letter signifies no statistical difference (ANOVA, *p* < 0.05).

Oxidative status of duckweeds exposed to salt and inoculated with *Pseudomonas* bacteria was analyzed by quantifying lipid peroxidation and hydrogen peroxide (Figure 4). Lipid peroxidation as a result of superoxide anion interacting with plant cells during oxidative stress was measured as malondialdehyde content (MDA). All inoculated duckweeds at all concentrations of NaCl had significantly less MDA content than their non-inoculated counterparts (Figure 4A). Hydrogen peroxide (H_2_O_2_) content was simultaneously measured to quantify this reactive oxygen species accumulated in duckweed tissue (Figure 4B). There was significantly more hydrogen peroxide in duckweeds inoculated with *P. oryzihabitans* D1-104/3 at 10 mM compared to 0 mM. At 100 mM, *P. gessardii* C31-106/3-inoculated duckweeds had more H_2_O_2_ than *P. oryzihabitans* D1-104/3-inoculated ones.

##### 2.3.1.3 Total polyphenol content and DPPH scavenging — Antioxidant capacity

**Figure 5:**
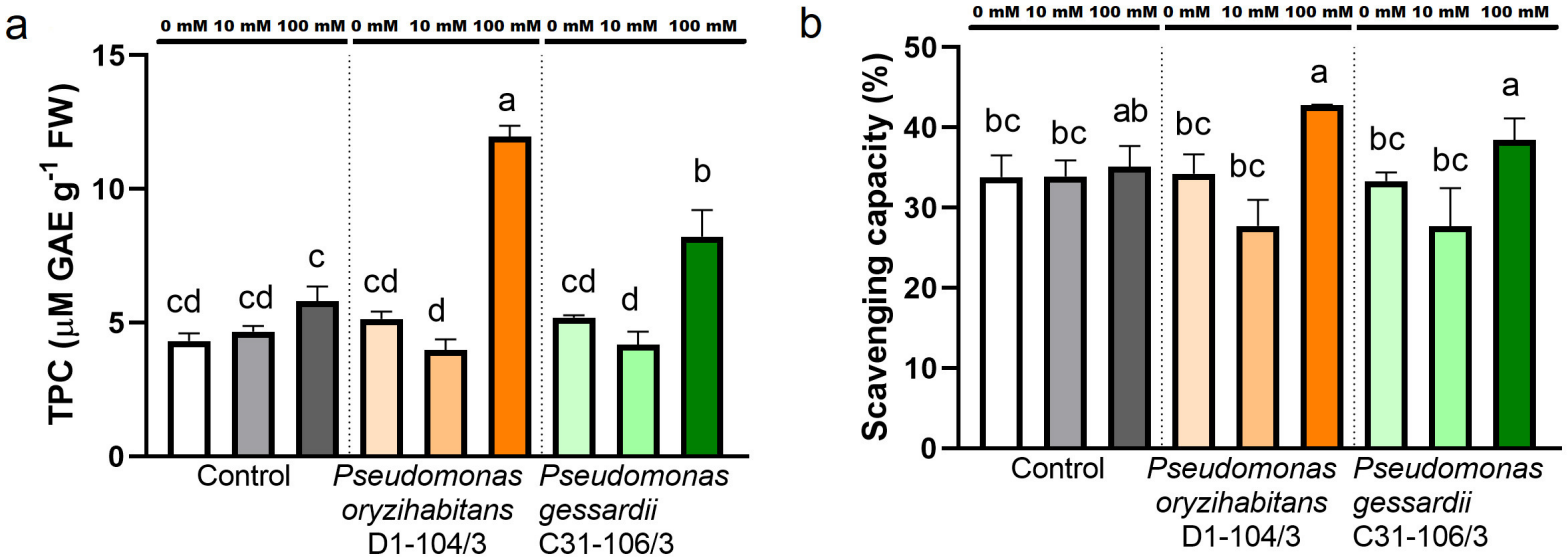
Total polyphenol content (TPC) and scavenging capacity of duckweeds at 0, 10 and 100 mM NaCl. **(A)** Total polyphenol content; **(B)** scavenging capacity. Orange columns—duckweeds inoculated with *P. oryzihabitans* D1-104/3. Green columns—duckweeds inoculated with *P. gessardii* C31-106/3. Bars represent the mean of three replicates, with standard errors. At least one same letter signifies no statistical difference (ANOVA, *p* < 0.05).

To assess the effects of salt and inoculation on polyphenols and DPPH scavenging capacity, these parameters were measured in parallel (Figure 5). Total polyphenol content (TPC) was significantly increased at 100 mM NaCl in inoculated duckweeds compared to surface-sterilized ones (Figure 5A). At 100 mM NaCl, the inoculated duckweeds had higher antioxidant capacity than at 0 and 10 mM (Figure 5B).

##### 2.3.1.4 Metabolites involved in response to salt stress: AsA, starch, and proline

**Figure 6:**
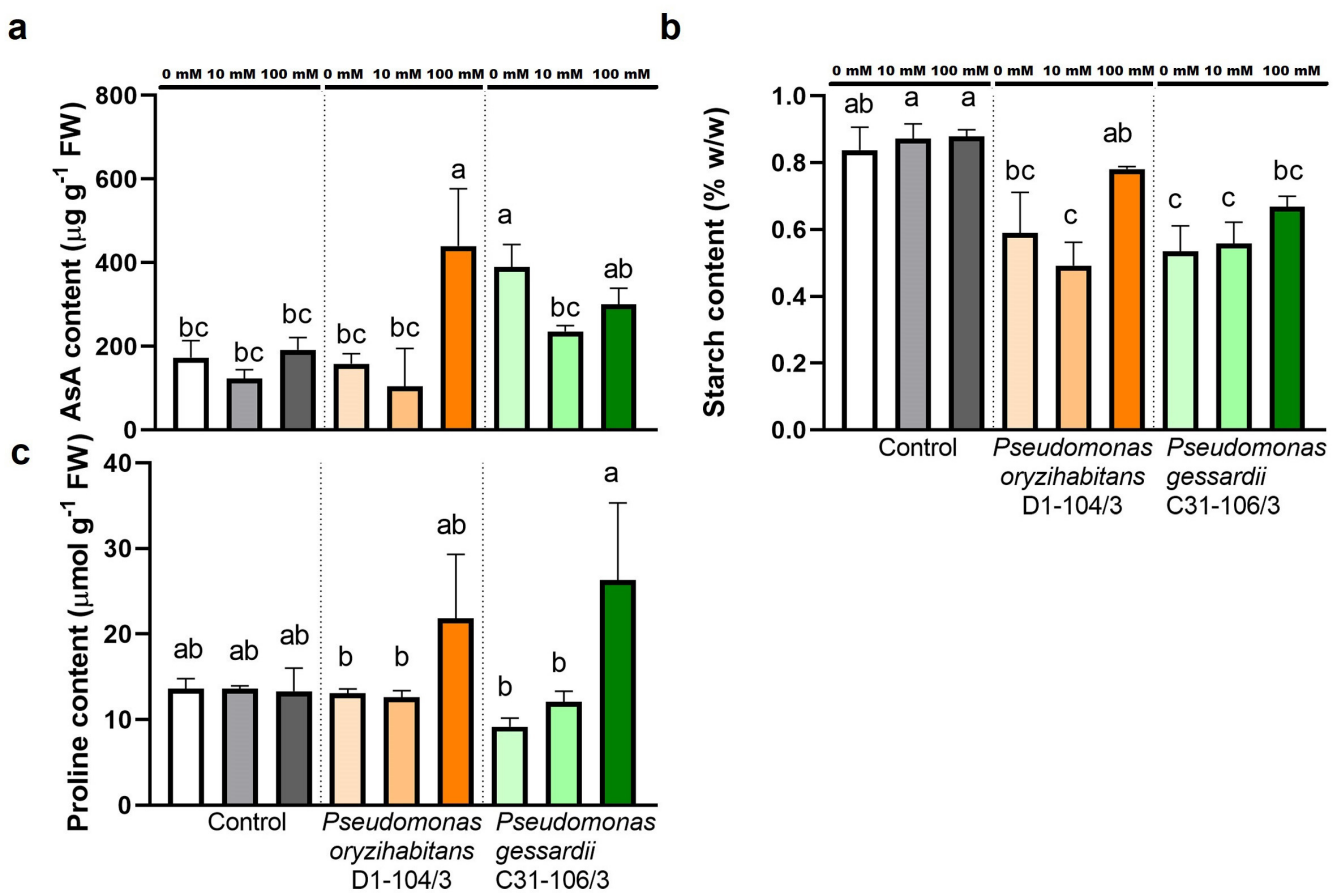
Metabolites involved in response to salt stress in duckweeds at 0, 10 and 100 mM NaCl. **(A)** ascorbic acid; **(B)** starch; **(C)** proline. Orange columns—duckweeds inoculated with *P. oryzihabitans* D1-104/3. Green columns—duckweeds inoculated with *P. gessardii* C31-106/3. Bars represent the mean of three replicates, with standard errors. At least one same letter signifies no statistical difference (ANOVA, *p* < 0.05).

Three important metabolites (AsA, starch, and proline) were quantified to assess the effects of salt treatment and bacterial inoculation on duckweeds (Figure 6). Ascorbic acid content was quantified due to its antioxidant potential in protecting plant cells from oxidative stress. The highest amount of AsA was measured in duckweed inoculated with *P. oryzihabitans* D1-104/3 at 100 mM and with *P. gessardii* C31-106/3 at 0 and 100 mM NaCl (≥300 μg g^−1^ FW). Duckweeds inoculated with *P. gessardii* C31-106/3 at 0 mM NaCl also had significantly more AsA content (Figure 6A).

Starch content was estimated from acidic extracts of duckweeds at the end of the 2-week experiments to assess effects of salt stress on carbohydrate metabolism. The results were presented as percentage of starch mass in fresh weight (% w/w). All inoculated specimens had significantly less starch content than non-inoculated ones, except for duckweeds inoculated with *P. oryzihabitans* D1-104/3 at 0 and 100 mM NaCl (Figure 6B).

Content of amino acid proline, an osmolyte, was quantified to analyze differences in effects of inoculation with two different *Pseudomonas* strains on duckweeds. The proline content remained mostly the same across treatment groups. Only duckweeds inoculated with *P. gessardii* C31-106/3 had significantly more proline at 100 mM than at 0 and 10 mM (Figure 6C).

#### 2.3.2 Total soluble proteins and enzymatic parameters (SOD, CAT, POX)

**Figure 7:**
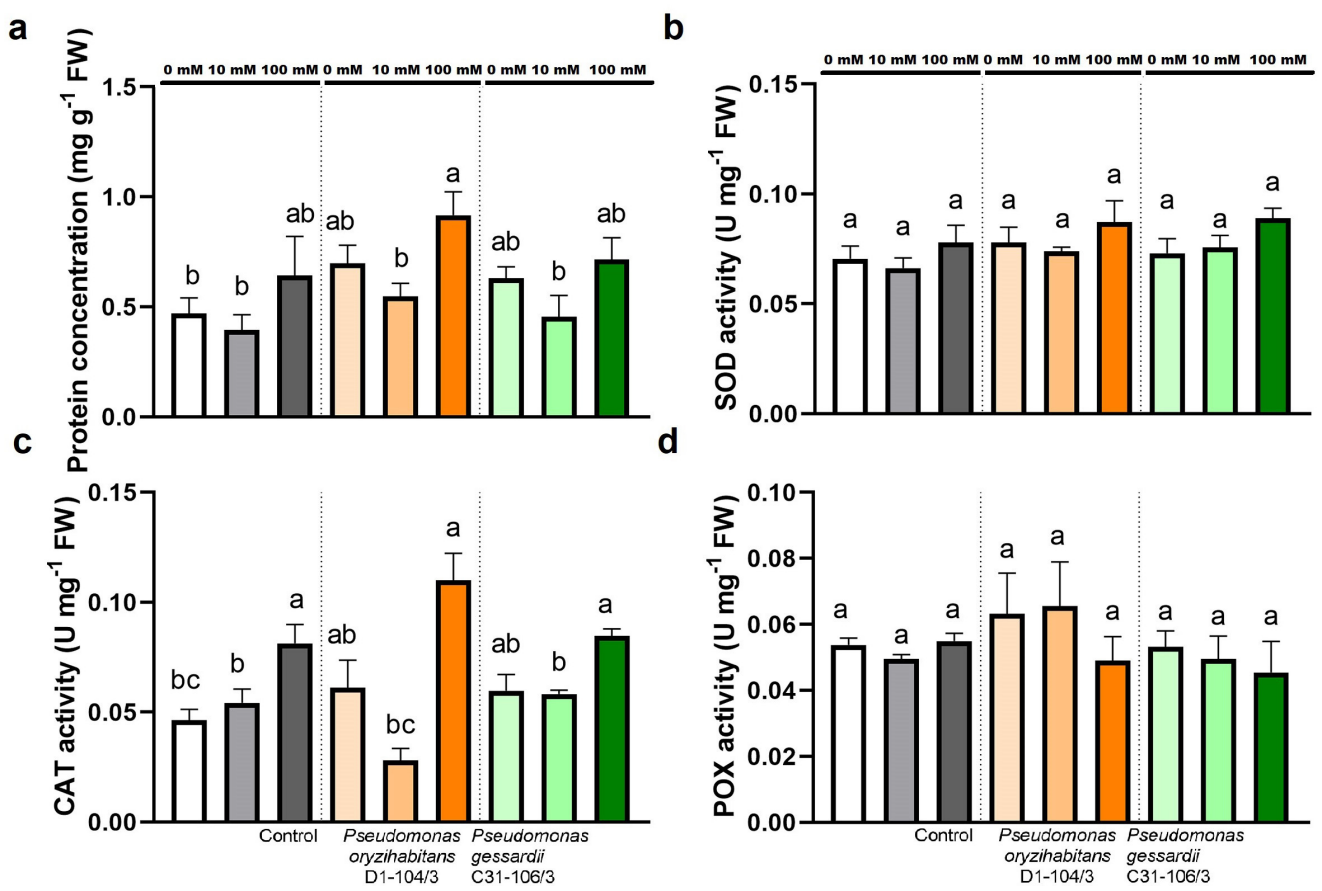
Total soluble proteins **(A)** and enzymatic activity (SOD, CAT, POX) **(B–D)** of duckweeds at 0, 10, and 100 mM NaCl. Orange columns—duckweeds inoculated with *P. oryzihabitans* D1-104/3. Green columns—duckweeds inoculated with *P. gessardii* C31-106/3. Bars represent the mean of three replicates, with standard errors. At least one same letter signifies no statistical difference (ANOVA, *p* < 0.05).

Total soluble proteins and enzymatic activity of SOD, CAT, and POX were measured to further assess the effects of salt treatment and bacterial inoculation on duckweeds (Figure 7). TSP were quantified to assess the effects of two *Pseudomonas* strains and salt stress on duckweeds' protein metabolism. The TSP remained mostly the same, except in duckweeds with *P. oryzihabitans* D1-104/3 at 100 mM, where it was significantly increased compared to 0 and 10 mM (Figure 7A).

The activities of central antioxidant enzymes: superoxide dismutases (SOD), catalases (CAT), and peroxidases (POX) were measured in order to analyze effects of inoculation and salt stress on duckweeds. Results showed that superoxide dismutase was equally active in all samples, regardless of inoculation and NaCl concentration (Figure 7B). Catalase activity was the highest at 100 mM in all samples (Figure 7C). There was no statistically significant difference in activities of total soluble peroxidases (POX) across all samples (Figure 7D).

### 2.4 Principal component analysis

**Figure 8:**
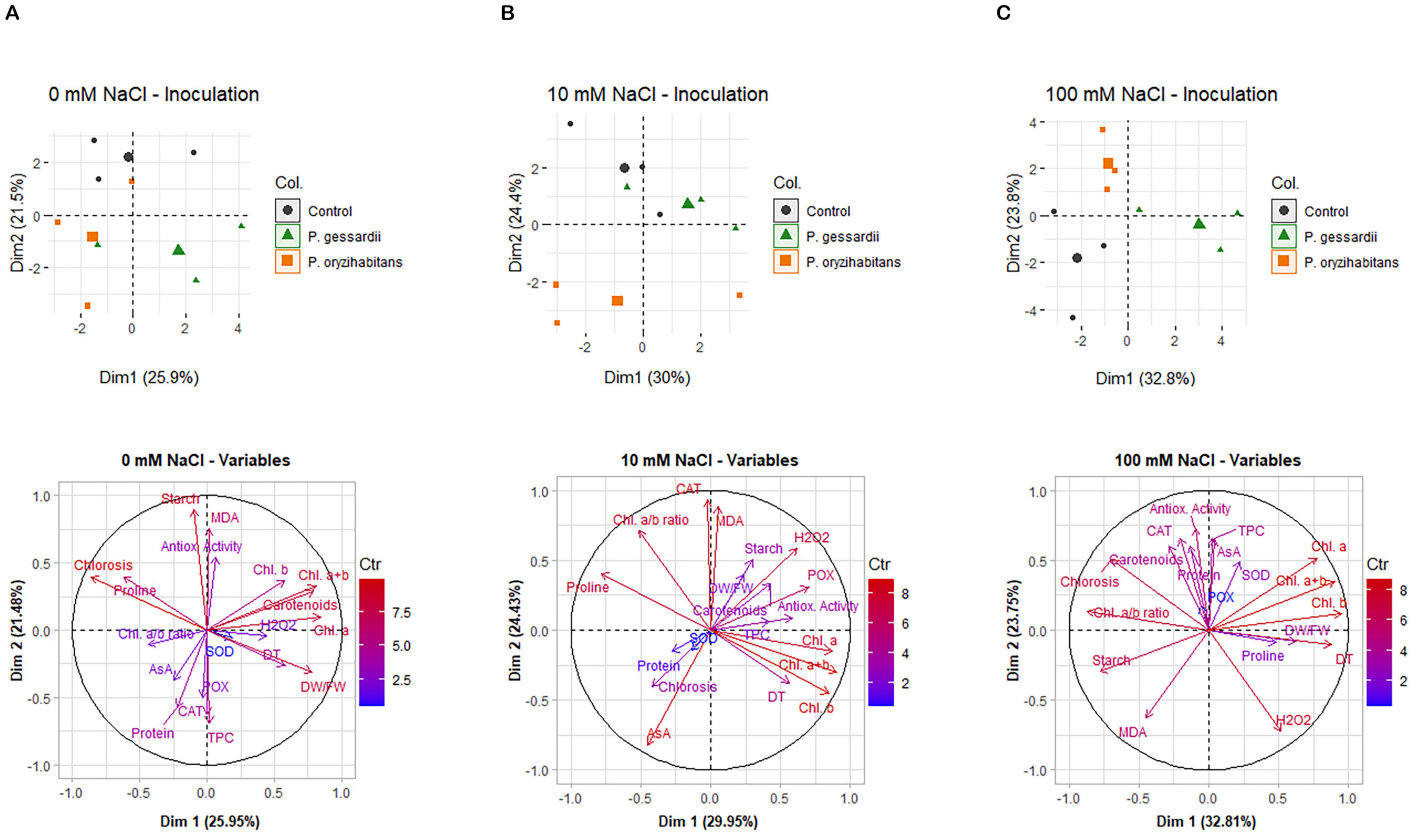
Principal component analysis (PCA) of data from the 0 mM NaCl **(A)**, 10 mM NaCl **(B)**, and 100 mM NaCl **(C)** treatments. The data distribution plots (upper row) and vector plots of variables (bottom row) were constructed in R, using the packages Factoextra and FactoMineR, respectively.

Principal component analysis was carried out for each of the three salinity levels: 0 mM (Figure 8A), 10 mM (Figure 8B), and 100 mM NaCl (Figure 8C). With increasing salinity levels, the relative contribution of principal component 1 (PC1) to total data variability increased from 25.9% at 0 mM NaCl to 30.0% at 10 mM NaCl, and finally, to 32.8% at 100 mM NaCl, suggesting an increasingly deterministic pattern of data distribution in the presence of stress, as has been already reported in literature (e.g., Napar et al., 2022).

At all the three salinity levels, the data corresponding to *P. gessardii* C31-106/3 are shifted along the x-axis when compared to the rest of the data, and this shift is particularly pronounced at 100 mM NaCl (Figure 8C). This suggests that the inoculation of *Lemna* with *P. gessardii* C31-106/3 resulted in a clear shift in the values of PC1, which accounts for the greatest share of the total data variability at each of the applied salinity levels. On the other hand, at all the three salinity levels, no shift along the *x*-axis was observed between the data corresponding to the non-inoculated (control) *Lemna* plants, and those inoculated with *P. oryzihabitans* D1-104/3. However, a pronounced shift between these two treatments was observed along the *y*-axis, especially at 10 and 100 mM NaCl, corresponding to the shift in PC2, the component of data variability with the second-greatest contribution to total data variability. Thus, the results of PCA suggest that, at each of the three salinity levels, *P. gessardii* C31-106/3 and *P. oryzihabitans* D1-104/3 affected the measured parameters in two different and mutually unrelated ways, with *P. gessardii* C31-106/3 accounting for overall more pronounced shifts in the values of the measured data (consistent with shifts in PC1), compared to *P. oryzihabitans* D1-104/3 (consistent with shifts in PC2).

## 3 Discussion

### 3.1 Localization of *Pseudomonas* bacteria on the duckweed root

It is well-known that the close physical interactions between PGPB and plants help the plants overcome the drought and salt stress (Gamalero and Glick, 2022). The localization pattern of bacteria on the root reported in this study may be explained by the release of root exudates, containing nutritious compounds such as amino acids, carbohydrates and phenolics, which act as chemoattractants (Mendes et al., 2013; Nordstedt and Jones, 2020; Yang et al., 2024). Interestingly, in our previous work we observed a different set of bacteria (*Klebsiella, Serratia, Hafnia*) also concentrated between root cells, albeit more around the midpoint of the root or elongation zone as opposed to the root tips and caps in this study, hinting at differences in ecology of different bacterial species (Radulović et al., 2020). It is also worth noting that we reported increased aggregation of superoxide anion and hydrogen peroxide especially on the roots (Popržen et al., 2024). This indicated a higher metabolic activity and possible co-localization with bacteria, which is partially confirmed in this study.

### 3.2 Biomarkers of interactions of *P. oryzihabitans* D1-104/3 and *P. gessardii* C31-106/3 with duckweed (*L. minor*) under salt stress

#### 3.2.1 The anti—chlorotic effect of *P. gessardii* C31-106/3

It has already been reported that different PGPB strains may significantly shorten the doubling times of duckweeds (which can be attributed to regulating a “developmental switch”, as we will discuss later) and increase their dry mass (Yamakawa et al., 2018; Khairina et al., 2020). Interestingly, in this study, *P. gessardii* C31-106/3 not only significantly slowed down the doubling of duckweeds at 100 mM, but also protected duckweeds against chlorosis at 0 and 10 mM. Chlorosis (literally, “the loss of green”), is commonly caused by lack of nutrients and ethylene overproduction by stressed plants (Shekhawat et al., 2023). The central mechanism by which PGPB reduce chlorosis in plants is ACC deaminase activity, which leads to removal of ethylene precursor, the 1-aminocyclopropane carboxylic acid (ACC) (Chen et al., 2022; Shekhawat et al., 2023). Various *Pseudomonas* strains (*P. fluorescens* YsS6, *P. stutzeri* A1501, *P. migulae* 8R6, *P. corrugata* DR3, *Pseudomonas* UW40) were reported to have PGP effects on tomato, rice, and vine grape, based on ACC deaminase activity (Orozco-Mosqueda et al., 2019; Duan et al., 2021; Shekhawat et al., 2023). However, in this study, both strains decreased the initial ACC concentration *in vitro* at the same rate: therefore, other factors probably influenced chlorosis (see Supplementary material). Chlorosis might also be associated with the differences in chlorophyll content. Namely, in duckweeds, the PSII and electron transport chain seem to be the most affected by salt treatment (Appenroth et al., 2021). By capturing the excess ROS and producing osmolytes e.g., proline and exopolysaccharide polimers, and by increasing the uptake of essential elements like nitrogen, which helps maintain the photosynthetic proteins and thylakoid membranes, the *P. gessardii* C31-106/3 might indirectly ameliorate chlorosis and improve chlorophyll content (Gavito et al., 2019; Kumar et al., 2020).

#### 3.2.2 The increase in doubling time and increase in dry weight induced by *P. gessardii* C31-106/3

The PGPB strains control a delicate balance between supporting further growth and stimulating stress tolerance: depending on the context, i.e., the type of host plant, type of stress, bacterial strain and experimental conditions, one will be prioritized over the other. In nature, if faced with adverse conditions, some duckweeds will temporarily halt vegetative reproduction, often in favor of synthesizing important cell constituents and accumulating nutrients e.g., starch. The starch in duckweeds builds up after the activation of an abscisic acid-dependent developmental switch which re-directs the carbohydrates synthesized during photosynthesis from vegetative growth to energy reserves (Appenroth et al., 2021). In this study, at 100 mM NaCl, duckweeds inoculated with *P. gessardii* C31-106/3 slowed down their doubling but also significantly increased their dry-weight-to-fresh-weight ratio, indicating that the energy was redirected from vegetative reproduction to metabolism. It can be hypothesized that this strain modulates the activity of the duckweed developmental “switch” or produces abscisic acid, as various rhizosphere and endophytic bacteria produce ABA (Lievens et al., 2017). Surprisingly, contrary to the expectations, this increase in dry-weight-to-fresh-weight ratio was not strongly correlated with accumulation of proteins or starch (see Supplementary material). A recent metabolomic study with *Spirodela polyrhiza*, the giant duckweed, revealed that a PGPB *Ensifer* sp. SP4 increased N metabolism and photosynthesis, but this increase moved toward doubling, not starch synthesis (Toyama et al., 2022). Our hypothesis is that *P. gessardii* C31-106/3 increases the relative dry weight by redirecting the energy into the synthesis of other compounds, e.g., pigments and proline, and other Osmoprotective compounds (trehalose, polyamines, glycine betaine, etc.) and possibly lipids (see Supplementary material), while improving the uptake of mineral elements (Kumar et al., 2020; Loudari et al., 2022).

#### 3.2.3 The starch accumulation in surface-sterilized duckweeds

Microbiome controls plant metabolism and vice versa—plant metabolites attract and “select” beneficial bacteria, while bacteria regulate plant metabolites that are useful for their own growth (Sitaraman, 2015; López-Farfán et al., 2019; Zboralski and Filion, 2023). The starch accumulation at all salinity levels in surface-sterilized duckweeds used in this study can be explained with nutritional deficiency, which is a known response of many duckweeds (Sree and Appenroth, 2022). This behavior of duckweed clones used in this study further confirmed the findings of Sree et al. (2015) that even within the same species, there is significant phenotypic plasticity: for some clones, salt stress is the trigger for starch accumulation, while for others it is nutrient deficiency. In the study of the *S. polyrhiza* and PGPB *Ensifer* sp. SP4, surface-sterilized *S. polyrhiza* deposited the starch, while the inoculated plants used their starch reserves, which was interpreted as a positive response (Toyama et al., 2022). In our study, inoculated duckweeds had lower amounts of starch, which can be explained with bacterial redirection of simple carbohydrates produced during photosynthesis: this effect was more pronounced with *P. gessardii* C31-106/3. Although the giant duckweed was not salt-stressed, a parallel with this study can be drawn: *P. gessardii* C31-106/3 mobilizes starch at all salinity levels, similarly to *Ensifer* sp. SP4 improves photosynthetic pigments, and at 100 mM, probably redirects this energy into biosynthesis of protective compounds. However, CO_2_ absorption rate, metabolomics, gene expression and comparative transcriptomics are needed to test whether this is the case.

#### 3.2.4 The non-enzymatic biomarkers of oxidative stress in interactions of duckweeds and *Pseudomonas* bacteria under salt stress

At all concentrations, bacteria significantly decreased lipid peroxidation. This can be explained by bacterial ability to neutralize existing superoxide anions and possibly by downregulating superoxide anion production in the host (Karpinska and Foyer, 2024). Similarly to superoxide anion, H_2_O_2_ is not only a reactive oxygen species, but also a signaling molecule (Huang et al., 2019). It is possible that this increase reflects the interactions between this strain and the plant under stress conditions, especially the colonization of the host plant by bacteria. It is worth noting that PGPB can induce a tolerable level of oxidative stress and still promote the duckweed growth (Ishizawa et al., 2017a). Moreover, both *Pseudomonas* strains improved the total polyphenol content and antioxidant capacity of duckweeds at 100 mM. Polyphenols are powerful antioxidants that capture excess ROS, while antioxidant test with DPPH reflects the overall ability of plants to combat oxidative stress, as it was demonstrated in rice (Singh et al., 2020). A similar effect was observed in studies with rice and other PGPB (Razzaque et al., 2019; Singh et al., 2020).

Ascorbic acid content was significantly increased at 100 mM NaCl in duckweeds inoculated with *P. oryzihabitans* D1-104/3, while *P. gessardii* C31-106/3 improved this parameter at 0 mM. Ascorbic acid is a strong low-molecular weight antioxidant, particularly important in drought- and salt-stressed crops where it protects photosynthetic apparatus from oxidative damage, and stimulates the biosynthesis of chlorophylls, e.g., in tomato (Chen et al., 2021). Abiotic stress such as drought or salt stress will deplete AsA content in plants by directly inhibiting its biosynthesis and by activating antioxidant enzymes that use ascorbic acid, while even exogenously applied AsA will alleviate the salinity-induced oxidative stress in the soybean (Seminario et al., 2017). Although connections between AsA and salt stress are still underresearched, it is known that different plants will activate different strategies to replenish the AsA depot. Proline was also significantly increased in duckweeds inoculated with *P. gessardii* C31-106/3 at 100 mM NaCl. It is generally understood that proline has multiple positive effects on plant physiology. Proline acts as an osmoregulatory protectant and is particularly important in responses to drought and salt stress by crops such as cucumber, maize, rice, legumes, and tobacco (El Moukhtari et al., 2020). Metabolism of proline affects electron transport chains in mitochondria and in chloroplasts, mitigating the effects of salt-induced oxidative stress (Carillo et al., 2008). Proline may also inhibit the ROS-mediated apoptosis by neutralizing excess ROS (Signorelli et al., 2014; Vujanovic et al., 2022). Moreover, proline produced by the host plant stimulates biological nitrogen fixation, particularly under stress conditions (Carillo et al., 2008). Multiple authors also reported that abiotic stress (drought) combined with plant-growth promoting bacteria increases proline content in crops more than abiotic stress alone (Signorelli et al., 2014; El Moukhtari et al., 2020; Vujanovic et al., 2022). In this research, 100 mM NaCl could be the critical concentration that triggers proline accumulation in response to the colonizing bacterium, *P. gessardii* C31-106/3. According to other researchers, only certain PGPB will have this effect under specific stress conditions (Signorelli et al., 2014; Vujanovic et al., 2022).

Overall, the effects of salt stress and two different *Pseudomonas* strains on non-enzymatic parameters of duckweeds in this study necessitate future molecular studies that would help elucidate molecular mechanisms behind them: transcriptomics in particular would help explain the differences outlined in this study. Moreover, mass spectrometry would help analyze the content of nutrient medium after co-cultivation, as well as chemical composition of duckweeds subject to different treatments.

#### 3.2.5 Effects of *P. oryzihabitans* D1-104/3 and *P. gessardii* C31-106/3 on antioxidant enzymes of duckweed (*L. minor*) under salt stress

The ability of PGPB to improve nitrogen utilization by the plants, especially under stress and nutrient scarcity, is one of their central mechanisms (Carillo et al., 2008; Toyama et al., 2022; Zboralski and Filion, 2023). In this study, TSP remained mostly constant regardless of the salt stress and inoculation. This may indicate that duckweeds in this study have efficient intrinsic mechanisms to protect their proteins from extensive damage. To assess the activity of antioxidant enzymes of duckweeds in this study, we chose three principal enzymes: SOD, CAT and POX. Although we recorded a significant decrease in lipid peroxidation in inoculated duckweeds, which suggested a possible increase in SOD activity, the SOD activity was the same in all samples. This suggested that bacterial SOD removed superoxide anions by activating its own SOD, although it cannot be excluded that bacteria upregulated plant's own antioxidant enzymes in the early phases of salt exposure: a similar effect was described by Toyama et al. (2022) and particularly by Ishizawa et al. (2017b) where SOD, APX, and CAT were upregulated by PGPB. Alternatively, the PGPB produce protective substances that defend electron transport chains in mitochondria and chloroplasts, where the bulk of superoxide anion is produced (Michalski et al., 2020; Karpinska and Foyer, 2024). This corresponds to some extent with observations from our previous study, where the SOD activity fluctuated depending on type of inoculum, time, and whether the nutrient medium was supplemented with indole-3-acetic acid (Popržen et al., 2024). The fact that activities of all three enzymes are lower in this study than in the previous one can be attributed to the fact that, with time, *Pseudomonas* bacteria and plants achieve an equilibrium. This equilibrium is also reflected in uniform density of bacteria on the surface of duckweeds (see Supplementary material). A similar observation was made by other authors (Ishizawa et al., 2017a). Catalase is one of the first and universal responders during salt stress in plants: some rice mutants with defective catalase genes are found to have a very low level of survival if exposed to salt, highlighting the vital importance of this enzyme in combating effects of salt stress (Razzaque et al., 2019). A similar observation was made with *Arabidopsis*: mutant plants with impaired CAT genes exhibit high levels of sensitivity to salt (Mhamdi et al., 2010). It seems that CAT are more efficient at high stress levels (which corresponds to our observations) and more important in specialized response to salt stress, while POX act more broadly in regulating homeostasis of the plants during salt stress (Mhamdi et al., 2010).

### 3.3 Summary

The results of the PCA analysis clearly showed that at all salt concentrations, compared to the control, both strains had distinct and separate effects on the plant physiology. However, the strain *P. gessardii* C31-106/3 appeared to have more beneficial effects, especially at the high salt concentration, where it impacts the stress-related variables more than *P. oryzihabitans* D1-104/3. This notion that abiotic stress has dose-dependent effects and that these are additionally regulated by PGPB has been reported by many other authors as well, in vastly different plants such as *Arabidopsis*, giant duckweed, rice, wheat, tobacco, maize, barley, peanut, and mungbean (Mhamdi et al., 2010; Cheng, 2011; Huang et al., 2019; Isayenkov and Maathuis, 2019; Ishizawa et al., 2019a,b; Kumar et al., 2020).

The results presented in this study show that both *Pseudomonas* strains improve various biochemical parameters of duckweeds and also suggest that *Pseudomonas* strains differ in their PGPB mechanisms: *P. gessardii* C31-106/3 prioritizes survival reflected in inhibition of vegetative reproduction, reduction of chlorosis, and increase in dry weight, while *P. oryzihabitans* D1-104/3 primarily supports expansion of duckweeds. The main questions stemming from this study are: (1) What are the molecular mechanisms by which these *Pseudomonas* strains control the metabolism and antioxidative response of duckweeds? (2) What are the mechanisms behind the anti-chlorotic effect, the slowing down of doubling time, and the increase in dry weight of duckweeds inoculated with *P. gessardii* C31-106/3? (3) Can these two strains be used simultaneously to improve duckweed growth? (4) Will these strains retain similar effects in the studies of response of other plant species to abiotic stress, e.g., drought?

## 4 Material and methods

### 4.1 Culture conditions

#### 4.1.1 Duckweed (*L. minor* L.)

Duckweed plants (*L. minor* L.) were surface-sterilized according to previously established protocol and kept in a stock Murashige-Skoog medium (MS) supplemented with 3% sucrose at 24 ± 2°C [under fluorescent light of 40 μmoL m^−2^ s^−1^ with 16 h light/8 h dark photoperiod (Popržen et al., 2023, 2024)]. The stock medium was replenished every month. For the purposes of the experiments, the duckweeds were cultured in MS medium without sucrose and vitamins (in further text: MS medium) for 14 days at same light and temperature conditions as mentioned above.

#### 4.1.2 *Pseudomonas* bacteria

The bacterial strains *P. oryzihabitans* D1-104/3 and *P. gessardii* C31-106/3 were selected from the pre-existing collection of rhizosphere-associated isolates (Radulović et al., 2019) based on their ability to produce and degrade IAA, and to regulate multiplication of duckweeds (Popržen et al., 2023). Cultures were activated from bacterial samples that were kept at −80°C in liquid Luria-Bertani (LB) medium with glycerol. Then, the bacterial cultures were transferred to solid LB medium and kept in refrigerator during the experiment. For inoculation of duckweeds, a single bacterial culture from each of the isolates was picked from LB agar and incubated overnight at 30°C and 220 rpm in a thermal shaker (IKA KS 4000 i control, Staufen, Germany) using liquid LB medium. Post-incubation, the bacterial cultures were centrifuged at 3,000 rpm for 10 min, washed with sterile MS medium, and then introduced to the flasks containing duckweeds in 50 ml of sterile MS medium at a ratio of 1:100 (v/v) for the initial bacterial density of ~10^8^ CFU ml^−1^. One flask contained either *P. oryzihabitans* D1-104/3 or *P. gessardii* C31-106/3.

### 4.2 Salt treatment and co-cultivation with *Pseudomonas* bacteria

Each salt treatment (10 mM and 100 mM sodium chloride in MS medium) as well as 0 mM NaCl were set as triplicates (three flasks per treatment with 50 ml MS medium each). Each treatment group, including 0 mM NaCl, was inoculated with either *P. oryzihabitans* D1-104/3 or *P. gessardii* C31-106/3 as described in the Section 4.1.2. Surface-sterilized duckweeds were used as control. For estimation of frond number, total frond area, and dry weight, an initial group of surface-sterilized (150 ± 50) duckweeds was relocated from the stock MS medium to MS medium. For studies of antioxidant parameters and chlorosis, the initial, surface-sterilized fresh plant material containing (500 ± 50) mg was placed in MS medium. At the end of the experiment, duckweed samples were photographed, their fresh weight was measured, then quickly frozen in liquid nitrogen, and kept at −20°C for analyses of antioxidant parameters.

### 4.3 Fluorescence microscopy of duckweeds and their interactions with *Pseudomonas* bacteria

The Live/Dead BacLight Bacterial Viability Kit (Molecular Probes-Thermo Fisher Scientific, San Diego, CA) for fluorescence staining was used to discriminate between dead and live bacteria on the duckweed surface. Briefly, duckweeds were randomly collected from each biological replicate (~6 individual duckweeds from each flask). These were immersed in 1 μl/ml solution of Live/Dead BacLight mixture according to manufacturer's specifications. The specimens were incubated at room temperature, in darkness, for 15 min. After that, stained duckweeds were transferred to microscopic slides, squashed, and mounted with BacLight microscopy oil. The microscopic preparations of at least three different plants were observed under fluorescence microscope (Leica, Wetzlar, Germany) and photographed. At least three fields of a single plant were inspected and the most informative fields (with the most organized and dense bacterial presence, evaluated visually) were presented.

### 4.4 Growth parameters of duckweeds inoculated with *Pseudomonas* bacteria during salt stress experiments

#### 4.4.1 Dry weight, relative growth rates (RGR), and chlorosis estimation

Dry weight of duckweeds was measured after drying in the oven at 60°C for 5 days. Frond numbers, total frond area and the extent of chlorosis (in percents) were estimated using free Java-based software ImageJ. Frond area was estimated as total area of the surface of culture medium covered with duckweeds. Chlorosis was estimated as white area vs. total area (white + green area). Relative growth rates were calculated based on frond numbers with the following equation:


(1) $$\begin{array}{l} \frac{RGR=\left(\ln\text{x}14-\ln\text{x}0\right)}{14} \end{array}$$

where x_14_ and x_0_ represent frond numbers on the 14th and the first day of the experiment, respectively.

### 4.5 Assessment of oxidative and antioxidative parameters of duckweeds

#### 4.5.1 Chlorophylls and carotenoid content

To determine chlorophylls and carotenoids in duckweed samples, ~20 mg of plant tissue from cultures was placed into 2 mL Eppendorf tubes filled with 2 mL of 96% ethanol (Zorka Pharma Hemija doo, Šabac, Serbia) and then incubated in a water bath (Univeba JP Selecta, Barcelona, Spain) at a temperature of 70°C for 10 min. The levels of chlorophylls and carotenoid present were determined using a UV-VIS spectrophotometer (Agilent 8453, Agilent Technologies, Santa Clara, CA, USA), with absorbance readings taken at wavelengths of 470, 648, and 664 nm. The concentrations of these pigments were computed based on the method outlined by Lichtenthaler, as referenced in Ðurić et al. (2023).

#### 4.5.2 Non-enzymatic parameters

##### 4.5.2.1 Lipid peroxidation and hydrogen peroxide (H_2_O_2_) content

To assess oxidative stress of the plants, total malondialdehyde (MDA) content as indicator of lipid peroxidation, as well as H_2_O_2_ content were quantified following previously established protocols, explained in detail in Ðurić et al. (2023). Briefly, to assess the presence of MDA (malondialdehyde), the method by Heath and Packer was applied as per our previous research (Popržen et al., 2024). In summary, 0.1 g of plant tissue was pulverized in liquid nitrogen using a pestle and mortar, and then dissolved in 0.1% trichloroacetic acid (TCA). This extract was centrifuged and the supernatant was combined with a solution containing 0.5% thiobarbituric acid (TBA) and 20% TCA. This combined solution was boiled and then quickly cooled on ice. Subsequently, the supernatant was used for spectrophotometric analysis at 520 and 600 nm wavelength. In parallel, to estimate H_2_O_2_ content, the same plant extract used for MDA detection was mixed with potassium iodide reagent in 10 mM potassium phosphate buffer, pH 7.0, and 1 M potassium iodide according to method by Velikova, also explained in Ðurić et al. (2023) and Popržen et al. (2024). The absorbance was read at 390 nm. Spectrophotometric readings of these and other non-enzymatic parameters were performed USING a Bio Tek Synergy H1 microplate reader, Agilent Technologies, Santa Clara, CA, USA.

##### 4.5.2.2 Total polyphenol content (TPC) and DPPH method (antioxidant capacity in plants)

The methods outlined by Ðurić et al. (2023) were used to determine the total polyphenol content and antioxidant capacity of duckweeds. Briefly, the quantification of total polyphenols was conducted based on Folin-Ciocalteu principles, whereas antioxidant capacity was determined with the use of the DPPH (1,1-diphenyl-2-picrylhydrazyl) technique. Plant ethanol extracts were combined with FC reagent and deionized water at room temperature following the Folin–Ciocalteu test (FC test). Solution (20%) Na_2_CO_3_ was added to the mixture after incubation, and it was then allowed to sit at room temperature for 90 min in the dark. The absorbance was measured at 765 nm. Gallic acid was used as a standard. For DPPH, the same ethanolic extracts used for the FC test were mixed with DPPH reagent, and dissolved in methanol. The samples were incubated at room temperature in the dark and absorbance was measured at 520 nm. The scavenging ability of DPPH radical was calculated as:


(2) $$\begin{array}{l} Inhibition\left(\%\right)=1-\left(Abs_{sample}-Abs_{control}\right)*100 \end{array}$$

##### 4.5.2.3 Ascorbic acid content

The level of ascorbic acid in control duckweeds (surface-sterile duckweeds in NaCl-free medium) and duckweeds treated with NaCl and/or inoculated with *Pseudomonas* strains was determined using the method described in Radulović et al. (2021). In brief, 0.1 g of fresh plant tissue was rapidly frozen in liquid nitrogen and then pulverized. This powder was then dissolved in 2 mL of 6% TCA solution and the ensuing extract was combined with 2% 2, 4 - dinitrophenylhydrazine and 10% thiourea, followed by heating in boiling water and immediate cooling on ice. The reaction was stopped by the addition of 85% sulfuric acid while on ice. Absorbance was measured at a wavelength of 530 nm on the microplate reader. The concentration of ascorbic acid in duckweeds was determined from a standard curve.

##### 4.5.2.4 Proline content

Total proline in duckweed samples was determined according to Carillo et al. (2008). Briefly, 0.1 g of fresh duckweed material was pulverized in liquid nitrogen and dissolved in 2 ml of 96% ethanol. The extracts were mixed with 1% ninhydrin reagent (ninhydrin dissolved in 60:20:40 glacial acetic acid, ethanol, and distilled water mixture), boiled in water bath at 95°C and quickly cooled on ice. The supernatant was used for spectrophotometric readings of Ruheman's purple, the chromogenic product of reaction with nynhidrin, with maximum absorbance at 570 nm. The concentration of proline was determined from the standard curve with known concentrations of proline standards.

##### 4.5.2.5 Starch content

Starch content of duckweeds was determined to assess the effects of inoculation and salt stress on carbohydrate metabolism, according to Sree et al. (2015). Fresh plant weight (200 mg) was homogenized in 4 milliliters of 18% (w/v) hydrochloric acid. After centrifuging the homogenate for 20 min at 5,000 g, it was shaken for 60 min at 5°C. The absorbance was measured spectrophotometrically in a cuvette at 605 and 530 nm after an aliquot of the diluted supernatant was combined with an equal volume of Lugol's solution (0.5% w/v KI and 0.25% w/v I_2_ in water). The amount of starch per fresh weight (S, in percentage w/w) was calculated according to the formula:


(3) $$\begin{array}{l} S=\frac{\left[C_{s}*Vol\left(extr\right)*100\right]}{FW} \end{array}$$

Where Cs is a coefficient calculated from known absorbances at 605 and 530 nm and absorbance coefficients as explained in detail in Sree et al. (2015).

#### 4.5.3 Protein extraction and enzymatic parameters

To assess activities of three antioxidant enzymes of duckweeds, protein extraction and spectrophotometric readings were performed as explained in greater detail previously. In summary, 1 gram of plant tissue was pulverized in liquid nitrogen. To this powder, a protein extraction buffer (1:1 v/w) consisting of Tris EDTA buffer (pH 8), glycerol, polyvinylpyrrolidone phosphate and protease inhibitors was added, as previously described (Popržen et al., 2024). The resulting mixture was then centrifuged. The supernatants were stored in aliquots at −20°C for subsequent analysis. The activity of superoxide dismutases (SOD, EC 1.15.1.1) was measured as described in Ðurić et al. (2023). The activity of peroxidases (POX, EC 1.11.1.7) was assessed following the method previously described in Ðurić et al. (2023) and Popržen et al. (2024). Catalase (CAT, EC 1.11.1.6) activity was determined using Aebi's protocol from 1984 with modifications, as described in Ðurić et al. (2023). Total protein concentrations were determined by Bradford method. The SOD activity was measured with the microplate reader, while the readings for kinetics of POX and CAT were taken using the cuvette spectrophotometer (Agilent 8453, Agilent Technologies, Santa Clara, CA, USA).

### 4.6 Statistical analysis

Each sample was set in three biological replicates (three flasks with MS medium containing multiple duckweeds). The bacteria—free group was set as control and was grown in MS medium with appropriate NaCl concentrations (0, 10, 100 mM). The results were expressed as mean ± standard error. Numerical data were analyzed in ANOVA, with *post-hoc* Duncan's test to separate the means at *p* < 0.05 significance level. Statistics were performed in STATISTICA software, version 8. For principal component analysis (PCA), the FactoMineR package (Lê et al., 2008) was run in R version 4.2.2 (R Core Team, 2022). FactoMineR was also used for the construction of vector plots of variables, whereas the package Factoextra (Kassambara and Mundt, 2020) was used for graphical representation of data distribution. All other graphs were generated in GraphPad Prism, Version 8 (StatSoft, Hamburg, Germany).

## Data Availability

The raw data supporting the conclusions of this article will be made available by the authors, without undue reservation.
