# Supplementary material for: Pseudomonas oryzihabitans D1-104/3 and P. gessardii C31-106/3 differentially modulate the antioxidative response of duckweed (Lemna minor L.) to salt stress
Source: Front Microbiol. 2024 Dec 18;15:1481437. doi: 10.3389/fmicb.2024.1481437 (PMC11688408; doi:10.3389/fmicb.2024.1481437)
Supplement: Supplementary file 1 [file Data_Sheet_1.docx]

Supplementary Material

# Estimation of *Pseudomonas* density on the duckweed surface

To estimate the surface density of *Pseudomonas* bacteria on duckweeds, the fresh plant material previously co-cultivated with either *P. oryzihabitans* D1-104/3 or *P. gessardii* C31-106/3 was macerated and diluted in sterile MS medium, and then streaked onto LB agar plates. The plates were incubated at +28⁰C for 3 days, and subsequently photographed and visually analyzed in *ImageJ* (Rueden *et al*., 2017). All dilutions were done in triplicates.

**2 Quantification of 1-aminocyclopropane-dexarboxylic acid (ACC)**

To assess the capacity of *Pseudomona*s strains to deactivate ethylene precursor, the ACC, the activity of ACC deaminase was indirectly quantified according to (Li *et al.*, 2011). Briefly, individual colonies in LB agar were picked and grown in 5 ml of LB medium overnight at 28°C with shaking at 200 rpm. Then, 2 ml of suspension were microcentrifuged at 8000 xg, 5 min. The pellet was washed and then resuspended in nitrogen-free Dworkin-Foster minimal medium and incubated overnight. The next day, the samples were briefly centrifuged and the pellet was resuspended in the medium containing ACC (3 mmol l^-1^) as the sole carbon source in a glass tube of 12 ml and incubated overnight. The samples were again briefly centrifuged, and the supernatant was diluted and used for the quantification of remaining ACC, using a standard curve with known concentrations of ACC. The absorbance was measured at 570 nm in the microplate reader. Statistically significantly lower concentration of ACC after incubation compared to sterile control with ACC was considered as evidence of bacterial ACC deaminase activity.

# 3 Microscopic estimation of total lipid content of duckweed inoculated with *P. gessardii* C31-106/3 at 100 mM NaCl

To visualize the presence of lipids in duckweeds inoculated with *P. gessardii* C31-106/3 at 100 mM NaCl, individual duckweeds were picked, cut into thin fragments, and stained with Sudan IV (Merck, Massachusetts, USA) according to (Uzelac *et al.*, 2015). In brief, the fragments were first immersed in 70% ethanol, and then transferred into 0.7% solution of Sudan IV in ethanol. The fragments were then rinsed in 50% ethanol and placed onto microscope slides with glycerol and coverslipped. The microscopic preparations were observed under the light microscope and photographed (Leica, Wetzlar, Germany).

# 4 Supplementary Figures


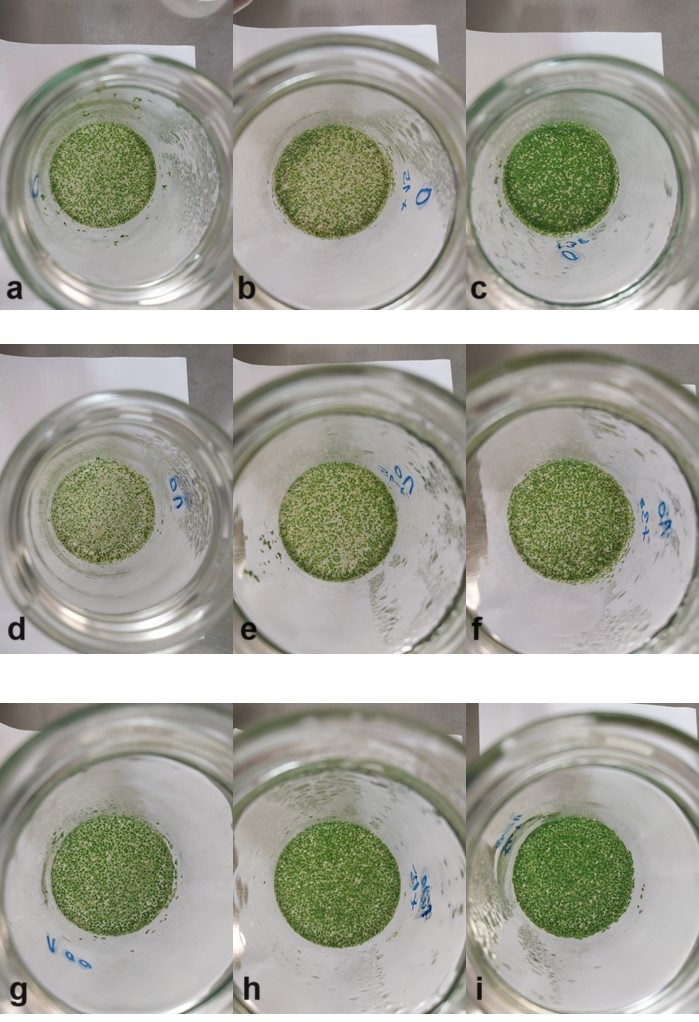


Supplementary Figure 1. Chlorosis. a, b, c – duckweeds at 0 mM: surface-sterilized (a); inoculated with *P. oryzihabitans* D1-104/3 (b); with *P. gessardii* C31-106/3 (c). d, e, f – duckweeds at 10 mM: surface-sterilized (d); inoculated with *P. oryzihabitans* D1-104/3 (e); with *P. gessardii* C31-106/3. (f). g, h, i - duckweeds at 100 mM: surface-sterilized (g); inoculated with *P. oryzihabitans* D1-104/3 (h); with *P. gessardii* C31-106/3 (i).


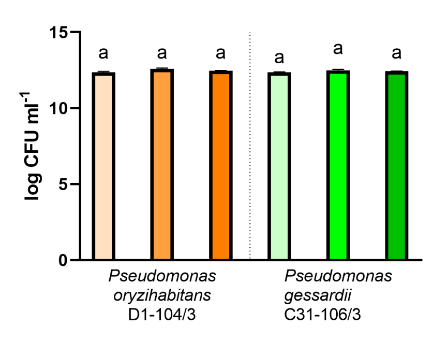


Supplementary Figure 2. Bacterial density on the surface of duckweeds. Orange columns – duckweeds inoculated with *P. oryzihabitans* D1-104/3. Green columns – duckweeds inoculated with *P. gessardii* C31-106/3. Bars represent the mean of 3 replicates, with standard errors. At least one same letter signifies no statistical difference (ANOVA, p < 0.05).


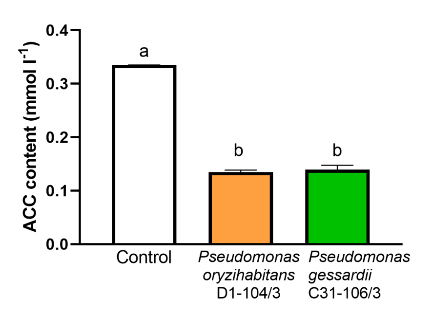


Supplementary Figure 3. ACC concentration. Control – sterile DF medium with ACC. Orange columns – duckweeds inoculated with *P. oryzihabitans* D1-104/3. Green columns – duckweeds inoculated with *P. gessardii* C31-106/3. Bars represent the mean of 3 replicates, with standard errors. At least one same letter signifies no statistical difference (ANOVA, p < 0.05).


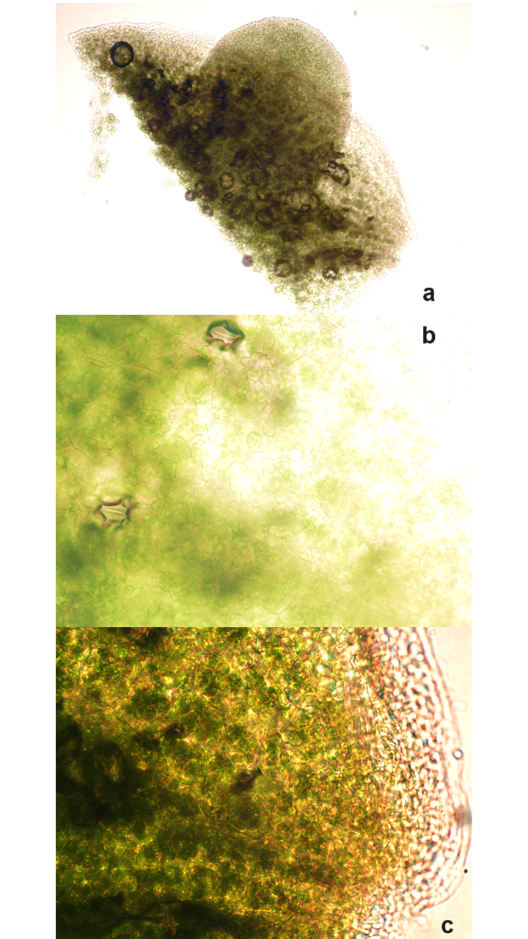


Supplementary Figure 4. Lipid content of duckweeds inoculated with *P. gessardii* C31-106/3 at 100 mM NaCl. a – a section of mother frond with budding daughter frond attached (10x). b – closed stoma; c – Sudan IV orange reaction with lipids in the cells of mother frond (40x).


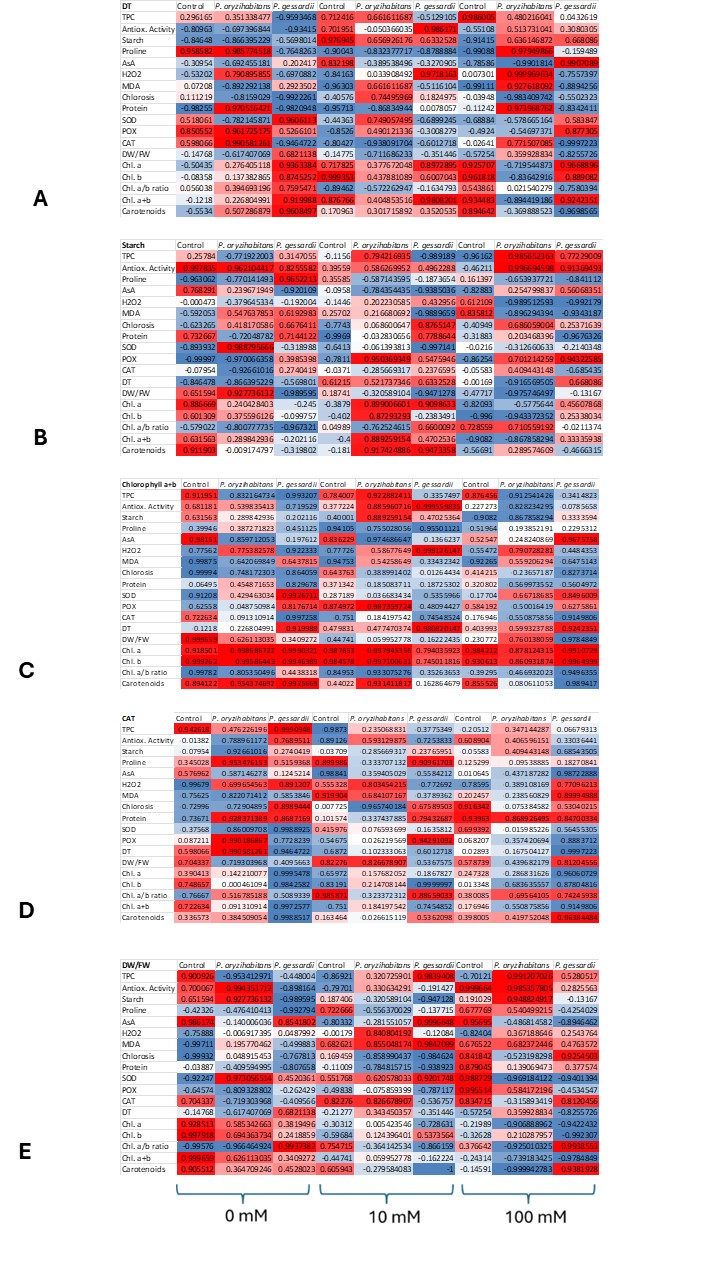


Supplementary Figure 5. Correlation coefficients between DT, starch, chlorophylls, CAT, DW/FW and other variables of duckweeds at three levels of salinity (0, 10, and 100 mM). The plants were inoculated with *P. oryzihabitans* D1-104/3 or *P. gessardii* C31-106/3. Control plants are surface-sterilized duckweeds at 0, 10 and 100 mM NaCl. The parameters were selected based on the statistically significant effect of bacterial strains on the said parameters. A – Doubling time; B – Starch; C – Chlorophyll a+b; D – Catalase; E – DW/FW.

**Supplementary References:**

Rueden, C.T., Schindelin, J., Hiner, M.C., DeZonia, B.E., Walter, A.E., Arena, E.T. and Eliceiri, K.W. (2017). ImageJ2: ImageJ for the next generation of scientific image data. BMC Bioinformatics, 18. DOI: [10.1186/s12859-017-1934-z](https://doi.org/10.1186/s12859-017-1934-z?form=MG0AV3)

Li, Z., Chang, S., Lin, L., Li, Y. and An, Q. (2011). A colorimetric assay of 1-aminocyclopropane-1-carboxylate (ACC) based on ninhydrin reaction for rapid screening of bacteria containing ACC deaminase. Letters in Applied Microbiology, 53, pp.178–185. DOI: [10.1111/j.1360-0443.2011.03592.x](https://doi.org/10.1111/j.1360-0443.2011.03592.x?form=MG0AV3)

Uzelac, B., Janošević, D., Stojičić, D. and Budimir, S. (2015). In vitro morphogenesis and secretion of secondary metabolites of *Nicotiana tabacum* tall glandular trichomes. Botanica Serbica, 39, pp.103-110. DOI: [10.1002/wdev.260](https://doi.org/10.1002/wdev.260?form=MG0AV3)
